# CXCR2-Driven Ovarian Cancer Progression Involves Upregulation of Proinflammatory Chemokines by Potentiating NF-κB Activation via EGFR-Transactivated Akt Signaling

**DOI:** 10.1371/journal.pone.0083789

**Published:** 2013-12-20

**Authors:** Yuan-Lin Dong, Syeda M. Kabir, Eun-Sook Lee, Deok-Soo Son

**Affiliations:** 1 Department of Biochemistry and Cancer Biology, Meharry Medical College, Nashville, Tennessee, United States of America; 2 Department of Physiology, Meharry Medical College, Nashville, Tennessee, United States of America; Yokohama City University School of Medicine, Japan

## Abstract

Ovarian cancer is an inflammation-associated malignancy with a high mortality rate. CXCR2 expressing ovarian cancers are aggressive with poorer outcomes. We therefore investigated molecular mechanisms involved in CXCR2-driven cancer progression by comparing CXCR2 positive and negative ovarian cancer cell lines. Stably CXCR2 transfected SKOV-3 cells had a faster growth rate as compared to control cells transfected with empty vector. Particularly, tumor necrosis factor (TNF), abundantly expressed in ovarian cancer, enhanced cell proliferation by decreasing the G0-G1 phase in CXCR2 transfected cells. TNF increased nuclear factor-κB (NF-κB) activity to a greater degree in CXCR2 transfected cells than control cells as well as provided a greater activation of IκB. CXCR2 transfected cells expressed higher levels of its proinflammatory ligands, CXCL1/2 and enhanced more proliferation, migration, invasion and colony formation. CXCR2 positive cells also activated more EGFR, which led to higher Akt activation. Enhanced NF-κB activity in CXCR2 positive cells was reduced by a PI3K/Akt inhibitor rather than an Erk inhibitor. CXCL1 added to CXCR2 positive cells led to an increased activation of IκB. CXCL1 also led to a significantly greater number of invasive cells in CXCR2 transfected cells, which was blocked by the NF-κB inhibitor, Bay 11-7082. In addition, enhanced cell proliferation in CXCR2 positive cells was more sensitive to CXCL1 antibody or an NF-κB inhibitor. Finally, CXCR2 transfection of parental cells increased CXCL1 promoter activity via an NF-κB site. Thus augmentation of proinflammatory chemokines CXCL1/2, by potentiating NF-κB activation through EGFR-transactivated Akt, contributes to CXCR2-driven ovarian cancer progression.

## Introduction

Ovarian cancer, one of several inflammation-associated cancers, is the fifth leading cause of cancer death among women. It is an insidious disease because it is typically asymptomatic until tumors have spread far beyond the ovaries [1]. The proinflammatory tumor microenvironment of ovarian cancer is clinically associated with peritoneal tumor dissemination and massive ascites, followed by a high mortality rate. Ovarian cancer cells express high levels of tumor necrosis factor (TNF), indicating the potential importance of TNF as a regulator of the proinflammatory tumor microenvironment in this malignancy [2]–[4]. Particularly, TNF has been shown to regulate chemokine networks in ovarian cancer cells through the nuclear factor-κB (NF-κB) signaling pathway [5]–[6]. Chemokines can be critical mediators in a tumor microenvironment by contributing to cancer progression and metastasis [7]–[8]. Among chemokine receptors, ovarian cancer cells frequently express CXCR2, which has prompted ovarian cancer progression [9]. CXCR2 is also highly expressed in certain other cancer cell types such as lung adenocarcinoma [10], laryngeal squamous cell carcinoma [11], endometrial carcinoma [12], rectal cancer [13], hepatocellular carcinoma [14] and gastric cancer [15]. Because of this association, it may be able to serve as an independent prognostic marker. Thus CXCR2 knockout mice have a significantly reduced tumor burden in prostate cancer [16], murine Lewis lung cancer [17] and renal tumor models [18] when compared to CXCR2 wild-type mice. In addition, a CXCR2 deficiency profoundly suppressed inflammation-driven tumorigenesis in skin and intestine [19]. The absence of CXCR2 in the tumor microenvironment also prevented colon cancer cell growth [20]. Finally, CXCL1, a CXCR2 ligand, was inversely associated with recurrence-free survival in colorectal cancer patients [21].

These facts indicate that a CXCR2-mediated signaling pathway is closely associated with cancer progression. Though multiple pathways such as apoptosis, EGFR activation and angiogenesis are involved in CXCR2-mediated signaling [9], [16]–[20], there is still a big gap on molecular mechanisms linking between CXCR2 and its multiple pathways. In our previous study, ovarian cancer cell lines highly expressed CXCL1-3 and CXCL8 [5]–[6] which all have a high affinity for CXCR2 [22]. Even though these CXCR2 ligands are tightly regulated by NF-κB signaling [5], [23], it is unclear how CXCR2 and NF-κB are mechanically involved in ovarian cancer progression. Here we used parental ovarian cancer cell lines and generated stable CXCR2 transfected cells as well as control cells transfected with empty vector. We then defined the impact of NF-κB signaling, a main proinflammatory pathway, on the potential contribution of CXCR2 to ovarian cancer progression.

## Materials and Methods

### Reagents

Recombinant human TNF, CXCL1 and a CXCL1/2/3 pan specific antibody for neutralization were obtained from R&D Systems (Minneapolis, MN). A human CXCL1/2 ELISA kit was purchased from PeproTech (Rocky Hill, NJ). PD98059 was purchased from EMD Chemicals Inc. (Gibbstown, NJ), AG-1478 was from Enzo Life Sciences International, Inc., (Plymouth Meeting, PA) and Bay11-7082 and LY294002 from Cayman Chemical (Ann Arbor, MI). Antibodies were purchased as follows: CXCR2 (E-2, sc-7304) and β-actin were from Santa Cruz Biotechnology (Santa Cruz, CA) and IκB, IKK, EGFR, Erk1/2, Akt and their phosphorylated forms, such as IκB (Ser32/36), EGFR (Tyr1173), Erk1/2 (Thr202/Tyr204), IKK (Ser176/180) and Akt (Ser473), were from Cell Signaling Technology (Beverly, MA). Lipofectamine 2000, G418 and all liquid culture media were acquired from Invitrogen (Grand Island, NY). A customized PCR array for the chemokine network, PCR array sets for cell-cycle related genes, a SYBR® Green Master Mix, and shRNAs for control and CXCR2 came from SABiosciences in Qiagen (Frederick, MD). Chemiluminescent detection kits were from GE Healthcare (Piscataway, NJ). Antisense and sense oligonucleotides were obtained from Eurofins MWG Operon (Huntsville, AL). CXCR2 expression vector was very kindly provided by Dr. Ann Richmond (Vanderbilt University, Nashville, TN) while the NF-κB luciferase vector came from BD Biosciences (Palo Alto, CA). The siRNAs for control and Akt1 were purchased from Cell Signaling Technology (Beverly, MA). Finally, the Luciferase Reporter Assay System, *Renilla*-Glo™ Luciferase Assay System and pRL-TK vector were obtained from Promega (Madison, WI).

### Stable CXCR2 Expressing Cell Line and Cell Cultures

The human ovarian cancer cell line SKOV-3 was purchased from the American Type Culture Collection (Manassas, VA). CXCR2 expressing cell lines were generated by stably transfecting CXCR2 or empty vectors into parental SKOV-3 ovarian cancer cells and selecting G418-resistant clones. Briefly, subconfluent cells were transfected with CXCR2 or empty vectors using lipofectamine 2000 and then treated with G418 to select drug-resistant clones. The treated cells were changed with new media every 3 days until G418-resistant clones appeared. The expression of CXCR2 protein in the selected clones was confirmed by Western blot and confocal imaging analysis. Because expression of CXCR2 in SKOV-3 cells is controversial (5, 9), we confirmed the absence or at most trace expression of CXCR2 at mRNA and protein levels in parental cells using PCR array, qRT-PCR, Western blot and confocal imaging analysis. The CXCR2 positive cell line was termed SKCXCR2, and the CXCR2 negative control cell line, SKA. Ovarian cancer cells (approximately 5×10^4^ cells/ml) were cultured at 37°C in a water-saturated atmosphere of 95% air and 5% CO_2_ in 24- or 6-well plates with RPMI medium with penicillin/streptomycin and 10% FBS. After an overnight culture to allow cellular attachment to the plates, the medium was removed and fresh medium without FBS was added to remove any effects of ingredients contained in sera. Treatments with the various agents are described in detail in Results.

### Western Blots

Cell lysates were prepared, fractionated on SDS-polyacrylamide gels and transferred to nitrocellulose membranes as previously described [6]. Blocking of nonspecific proteins was performed by incubation of the membranes with 5% nonfat dry milk in Tris buffered saline Tween-20 (TBST) for 2 h at room temperature. Blots were incubated with primary antibodies at 1∶1,000 dilution in blocking solution overnight at 4°C. The membranes were washed 3 times with TBST for 10 min, followed by incubation for 1 h with horseradish peroxidase conjugated secondary antibody (1∶2,500 dilution) in 5% milk/TBST. The membranes were then rinsed 3 times with TBST for 10 min and the bands visualized by enhanced chemiluminescence. After membrane stripping for 10 min with methanol containing 3% H_2_O_2_, β-actin was detected in order to serve as an internal loading control of cell lysates.

### Cell Proliferation Assays

Cell proliferation assays were performed using the cleavage of 3-(4,5-dimethylthiazol-2-yl)-2,5-diphenyltetrazolium bromide (MTT) to a colored product. After incubation in a 24-well plate, each well was washed twice with phosphate-buffered saline (PBS) and then an MTT solution (1 mg/ml of phenol red-free media:PBS  =  4∶1) was added. The plates were incubated for 3 h with protection from light. The MTT solution was removed and 500 µl of isopropanol was added. The plates were placed on a shaker for 10 min at room temperature to thoroughly dissolve the MTT color product. Optical density was measured at 595 nm using a microplate reader (Bio-Rad, Hercules, CA). Values were normalized to untreated controls.

### Flow Cytometry

Cancer cells were seeded at equal densities and maintained in culture for 24 h. Cells were then treated in triplicate with TNF (10 ng/ml) or media alone as a control for 48 h. Adherent and nonadherent cells were harvested with cold PBS and stained with propidium iodide [50 mg/ml in 0.1% (w/v) sodium citrate, 0.1% (v/v) Triton X-100] overnight. After overnight incubation, samples were analyzed using a FACScan flow cytometer (BD Biosciences) and the percentage of cells in G0/G1, G2/M and S phases quantified utilizing FloJo software (Tree Star Inc., Ashland, OR).

### Transient Transfections and Luciferase Assays

CXCL1 promoter activity was performed with generated KC701LUC vector and its mutants as previously described [23]. Ovarian cancer cells at approximately 50% confluency in 24-well plates were washed once with fresh media without additives and then transiently transfected with target vectors or Akt1 siRNA (final concentration: 10 nM) for 24 h at 37°C using Lipofectamine solution. Transfected cells were treated as outlined in Results and incubated for 6 h. After rinsing cells with cold PBS and adding lysis buffer (Promega, Madison, WI), cell lysates were used for determination of luciferase activity using a microplate luminometer. Luciferase activity, expressed as relative light units, was normalized to measured protein levels or the activity of each internal control.

### Confocal Imaging Analysis

Cells (5000 cells/250 µl media) were seeded on 8-chambered slides and cellular attachment was allowed overnight. The cells in the chamber slides were washed 3 times in PBS and fixed with 4% paraformaldehyde for 10 min at room temperature and blocked with 1% BSA in PBS for 30 min. The primary antibody was applied for 1 h at room temperature, and then washed with PBS for 30 min. The slides were washed 3 times with PBS and then incubated with the second antibody conjugated with Alexa Fluor 594 or Alexa Fluor 488 (LI-COR Biotechnology, Lincoln, NE) for 1 h at room temperature. Finally, the slides were washed 3 times with PBS, mounted with mounting medium containing DAPI (Vector laboratories, Burlingame CA) and observed with a fluorescence microscope (Nikon A1R laser scanning confocal imaging).

### PCR Array and qRT-PCR

After isolating total RNA and eliminating genomic DNA, the RT reaction was performed at 42°C for 15 min followed by 94°C for 5 min. A real-time PCR reaction for cell-cycle related genes or chemokines was performed according to manufacturer’s instructions using a Bio-Rad CFX96 (Hercules, CA) and the following two-step cycling program: 1 cycle at 95°C for 10 min, and 40 cycles at 95°C for 15 sec and at 60°C for 1 min. Data analysis was performed based on a Web-Based PCR Array Data Analysis (http://pcrdataanalysis.sabiosciences.com/pcr/arrayanalysis.php) provided by SABiosciences in Qiagen (Frederick, MD). Primers used in qRT-PCR were as follows: 5-TGC AGG GAA TTC ACC CCA AG-3 (forward) and 5-GGA TGC AGG ATT GAG GCA AG-3 (reverse) for CXCL1 and 5-GCA GGG AAT TCA CCT CAA G-3 (forward) and 5-GGG GTT GAG ACA AGC TTT C-3 (reverse) for CXCL2.

### Enzyme-linked Immunosorbent Assay (ELISA)

Human CXCL1/2 activity was measured by a human CXCL1/2 ELISA kit (PeproTech, Rocky Hill, NJ) according to the manufacturer’s instructions. The optical density of each well was determined, using a microplate reader set to 450 nm with wavelength correction at 570 nm.

### Migration and Invasion Assays

Tumor cells (2X10^5^ cells/ml in serum-free RPMI-1640 medium with 1% BSA) to be used for a migration or invasion assay were seeded in the 24-well Transwell cell culture insert (Greiner Bio-one) or in Matrigel (BD Biosciences, 1∶3 diluted with PBS) coated Transwell system, respectively. The bottom chamber contained 0.5 ml RPMI supplemented with 10% fetal bovine serum as a chemoattractant. Cells were treated as indicated in Results and then incubated for 24 h. The cells that remained inside the insert were removed with a cotton swab; migrating or invading cells on the filter were fixed with 3.7% formaldehyde and stained with 0.1% crystal violet followed by washing of the cells with PBS. The number of migrating or invading cells was counted under the microscope (X400) using 5 randomly chosen fields.

### Colony Formation

Cells were suspended in RPMI medium containing 0.4% agarose at concentrations of 2×10^3^ cells per well of a six-well plate. The suspended cells were overlaid onto a bottom layer of solidified 0.8% agarose in RPMI medium with 5% FBS, and incubated for 14 days. Colonies were stained with 0.05% crystal violet, photographed, and quantified.

### Knockdown of CXCR2 by CXCR2 shRNA

Ovarian cancer cells at approximately 50% confluency in 24- or 6-well plates were washed once with 1% FBS fresh media without additives and then transiently transfected with Control or CXCR2 shRNA (final concentration: 1 µg/ml) for 72 h at 37°C using Lipofectamine solution. Transfected cells were confirmed knockdown of CXCR2 protein and treated as outlined in Results according to various experiments.

### Statistical Analysis

Data were analyzed by the paired Student’s *t*-test and one-way analysis of variance (ANOVA) as appropriate. If a statistical significance (p≤0.05) was determined by ANOVA, the data were further analyzed by Tukey’s pairwise comparisons to detect specific differences between treatments.

## Results

### CXCR2 Positive Cells Have a Faster Growth Rate and Are More Responsive to TNF-stimulated Cell Proliferation Compared to CXCR2 Negative Cells

**Figure 1:**
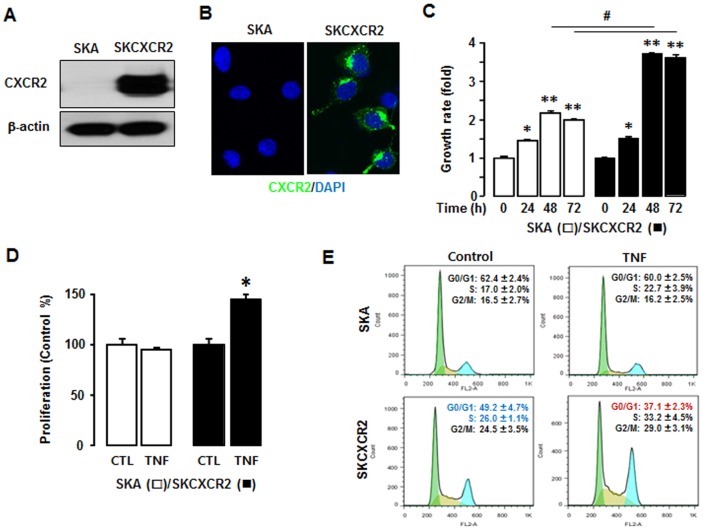
TNF enhances cell proliferation in CXCR2 positive cancer cells. (A) CXCR2 protein expression in SKA versus SKCXCR2 cells. Whole cell lysates were prepared and western blots carried out using antibodies specific to CXCR2 and β-actin as a loading control. (B) Representative immunofluorescent staining of SKA versus SKCXCR2 cells, indicating CXCR2 protein expression levels (in green). (C) Comparison of growth rates in SKA versus SKCXCR2 cells. Cells were incubated for 0, 24, 48 and 72 h and growth rates normalized to 0 h densities in each cell line. Experiments were performed in triplicate and all data are shown as mean ± S.E. * and ** (p≤0.05) in each group by ANOVA and Tukey’s pairwise comparisons. # (p≤0.05) between SKA and SKCXCR2 cells by the paired Student’s *t*-test. (D) Effect of TNF on cell proliferation in SKA versus SKCXCR2 cells. Cells were incubated with vehicle (Control) or TNF (10 ng/ml) for 48 h. A cell proliferation assay was performed using MTT and values normalized to untreated controls. Experiments were performed in triplicate and all data are shown as mean ± S.E. *(p≤0.05) by Student’s *t*-test. (E) TNF effects on cell cycle stages G0-G1, S and G2-M in SKA versus SKCXCR2 cells. Cells were treated with vehicle (Control) or TNF (10 ng/ml) for 48 h. Flow cytometry assays were performed to determine the % of cells in each phase. Representative histograms are shown. Experiments were conducted 5 independent times and data in each insert are shown as mean ± S.E. Blue and red letters indicate significance (p≤0.05) as compared to SKA control and TNF treatment, respectively, by Student’s *t*-test.

We generated CXCR2 positive (SKCXCR2) and negative (SKA) cell lines by stably transfecting CXCR2 or empty vectors into parental SKOV-3 ovarian cancer cells (Figures 1A and 1B). Growth rates in SKCXCR2 and SKA cells were similar for the first 24 h of culture but by 48 and 72 h, SKCXCR2 cell growth rates were roughly double as compared to SKA cells (Figure 1C). Since TNF is well known to be a proinflammatory cytokine abundantly expressed in ovarian cancer [2]–[4], we tested the effects of TNF on cell proliferation in SKA and SKCXCR2 cells. The results showed that TNF significantly increased cell proliferation in SKCXCR2 cells, but had no effect on the proliferation of SKA cells (Figure 1D). Based on FACS analysis, SKCXCR2 cells had a reduced G0-G1 phase and an increased S phase (with a slight increase in the G2-M phase) compared to SKA cells (Figure 1E). TNF per se, however, clearly decreased SKCXCR2 G0-G1 phase (with a slight increase in the S and G2-M phases) whereas it had no effects on SKA cells (Figure 1E).

In addition, we tested the effects of TNF on cell cycle-related genes in SKA versus SKCXCR2 cells. SKCXCR2 cells had above 50% decrease in cyclin B1 (0.49), cyclin F (0.38), cyclin G2 (0.47) and p21 (0.25) when compared to SKA cells. TNF had no significant effect on cell cycle-related genes in Control SKA cells, but it resulted in > 2 fold increase of GADD45α in SKCXCR2 cells (Table S1). GADD45α has been shown to be a mediator of synthetic retinoid induced apoptosis in ovarian carcinoma cells [24]. Thus disruption of GADD45α has been shown to promote tube formation and the migration of endothelial cells [25]. Cell migration and invasive abilities were indeed much higher in GADD45α-deficient mouse embryonic fibroblasts [26]. Based on these functional characteristics of GADD45α, a TNF-induced increase in GADD45α is unlikely to be associated with the enhanced cell proliferation in SKCXCR2 cells.

### CXCR2 Positive Cells Enhance NF-κB Activation Followed by an Increase of CXCR2 Ligands (CXCL1 and 2), as Compared to CXCR2 negative Cells

**Figure 2:**
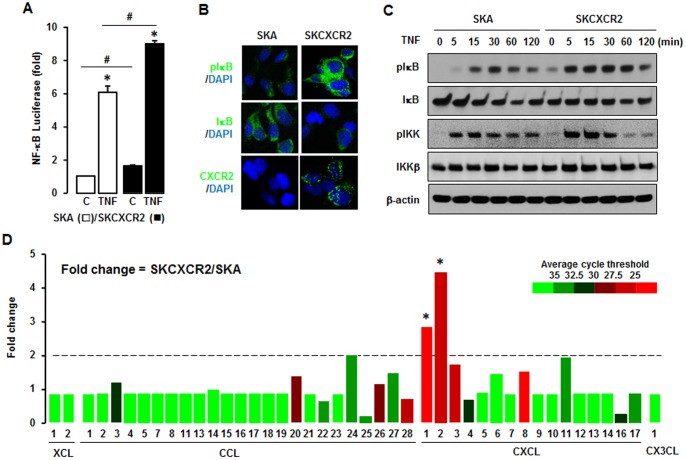
CXCR2 expressing cells have a higher activation of NF-κB in both basal and TNF-stimulated levels and increase CXCL1/2. (A) Effect of TNF on NF-κB luciferase activities in SKA and SKCXCR2 cells. After transfection using NF-κB luciferase vector overnight, cells were treated with TNF (10 ng/ml) for 4 h. Experiments were performed in triplicate and data are shown as mean ± S.E. * and # (p≤0.05) when compared to Control and SKA cells, respectively, by the paired Student’s *t*-test. (B) Representative immunofluorescent staining of SKA and SKCXCR2 cells indicating IκB activation and CXCR2 protein expression levels (in green). (C) Effect of TNF (10 ng/ml) over time (0–120 min) on NF-κB activation in SKA and SKCXCR2 cells. Whole cell lysates were prepared and Western blots carried out using antibodies specific to IκB and IKK as well as their phosphorylated forms (pIκB and pIKK). β-actin was used as a loading control. (D) Chemokine profile comparisons in SKCXCR2 relative to SKA cells. After isolating total RNA, a human chemokine PCR array was performed. The dotted line indicates a 2-fold increase; those with a >2-fold increase and average cycle threshold <30 are recognized as induced chemokines, and in this case represent CXCL1 and 2 (*).

As NF-κB is the primary signaling pathway for TNF functions, we therefore investigated if the TNF-induced cell proliferation in SKCXCR2 cells involved NF-κB signaling. Both basal and TNF-induced levels of NF-κB promoter activity were higher in SKCXCR2 cells (Figure 2A). Immunofluorescent staining revealed that SKCXCR2 cells had more phosphorylated IκB as compared to SKA cells (Figure 2B). On the other hand, IκB expression was higher in SKA cells as compared to SKCXCR2 cells (Figure 2B). Western blot analysis demonstrated that CXCR2 expressing cells had more phosphorylated IKK and IκB (as a direct downstream effect of IKK) in both their basal states as well as in response to TNF over time (Figure 2C). CXCR2-mediated NF-κB activation may involve chemokine ligands which contain κB sites in their promoters [5]–[6], [23]. Therefore we compared the chemokine network profiles in SKA and SKCXCR2 cells using a PCR array. The results showed that SKCXCR2 cells had a >2 fold increase in proinflammatory chemokines CXCL1 and CXCL2 when compared to SKA cells (Figure 2D).

### CXCR2 Positive Cells Increase CXCL1/2, and are Involved in Cell Proliferation and Enhance Migration, Invasion and Colony Formation Compared to CXCR2 Negative Cells

**Figure 3:**
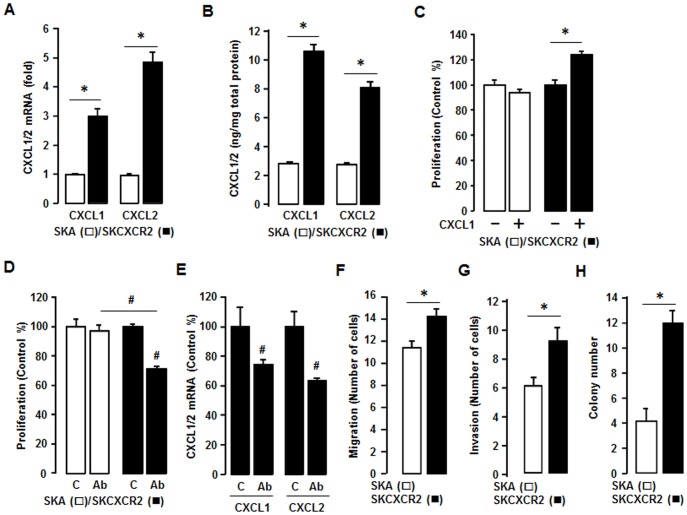
SKCXCR2 cells increases CXCL1/2, thus contributing to cell proliferation and enhancing migration, invasion and colony formation relative to values in SKA cells. (A) Confirmation of CXCL1 and CXCL2 expression in SKA and SKCXCR2 cells by qRT-PCR. After isolating total RNA, qRT-PCR was carried out using primers for CXCL1 and CXCL2. (B) Cellular CXCL1 and CXCL2 concentrations in SKA and SKCXCR2 cells over a period of 24 h. Whole cell lysates were prepared and ELISA carried out using antibodies specific to CXCL1 and CXCL2 and values were normalized to total protein. (C) Effect of CXCL1 on cell proliferation in SKA and SKCXCR2 cells for 48 h incubation. (D) Effect of pan antibody for CXCL1/2/3 on cell proliferation in SKA and SKCXCR2 cells. Cells were incubated with normal IgG (Control) and pan antibody (1∶100 dilution) for 48 h. The cell proliferation assay was performed using MTT and values were normalized to untreated controls. (E) Effect of pan antibody for CXCL1/2/3 on CXCL1 and CXCL2 expression in SKCXCR2 cells by qRT-PCR. After treating with pan antibody for 24 h and then isolating total RNA, qRT-PCR was carried out using primers for CXCL1 and CXCL2. (F) Migration characteristics between SKA and SKCXCR2 cells. (G) Invasion characteristics between SKA and SKCXCR2 cells. (H) Comparison of colony formation between SKA and SKCXCR2 cells. All experiments were performed at least in triplicate and data are shown as mean ± S.E. * and # (p≤0.05) as calculated by Student’s *t*-test.

We confirmed that SKCXCR2 cells produced more CXCL1 and CXCL2 than SKA cells by qRT-PCR and ELISA assay (Figures 3A and 3B). Although SKCXCR2 cells had a larger increase in CXCL2 than CXCL1 at the mRNA level (Figure 3A), as far as total protein, there was more total CXCL1 protein (Figure 3B) than CXCL2, probably resulting from higher amount of CXCL1 mRNA (Figure 2D). Based on the presumed CXCR2-NF-κB-CXCL1/2 connection, we tested if CXCL1 or its antibody affected cell proliferation differently in SKA and SKCXCR2 cells. Addition of CXCL1 had no effect on cell proliferation in SKA cells but significantly increased the proliferation of SKCXCR2 cells (Figure 3C). Also while a pan antibody for CXCL1/2/3 had no effect on cell proliferation in SKA cells it significantly decreased proliferation in SKCXCR2 cells (Figure 3D). Additionally we confirmed that the pan antibody reduced CXCL1 and CXCL2 mRNA in SKCXCR2 cells (Figure 3E). Because the CXCL1-CXCR2 axis was found to promote gastric tumor invasion [15], we compared the migration and invasion capabilities of SKA and SKCXCR2 cells. SKCXCR2 cells had enhanced migration and invasion properties compared to SKA cells (Figures 3F and 3G). Based on the increased migration and invasion in SKCXCR2 cells, we further tested a soft agar colony formation to detect if there were a higher malignant transformation in SKCXCR2 cells, and found that SKCXCR2 cells produced more colonies than SKA cells (Figure 3H).

### CXCR2 Positive Cells Transactivate EGFR to a Higher Degree, Resulting in Akt Activation Which Contributes to NF-κB Signaling

**Figure 4:**
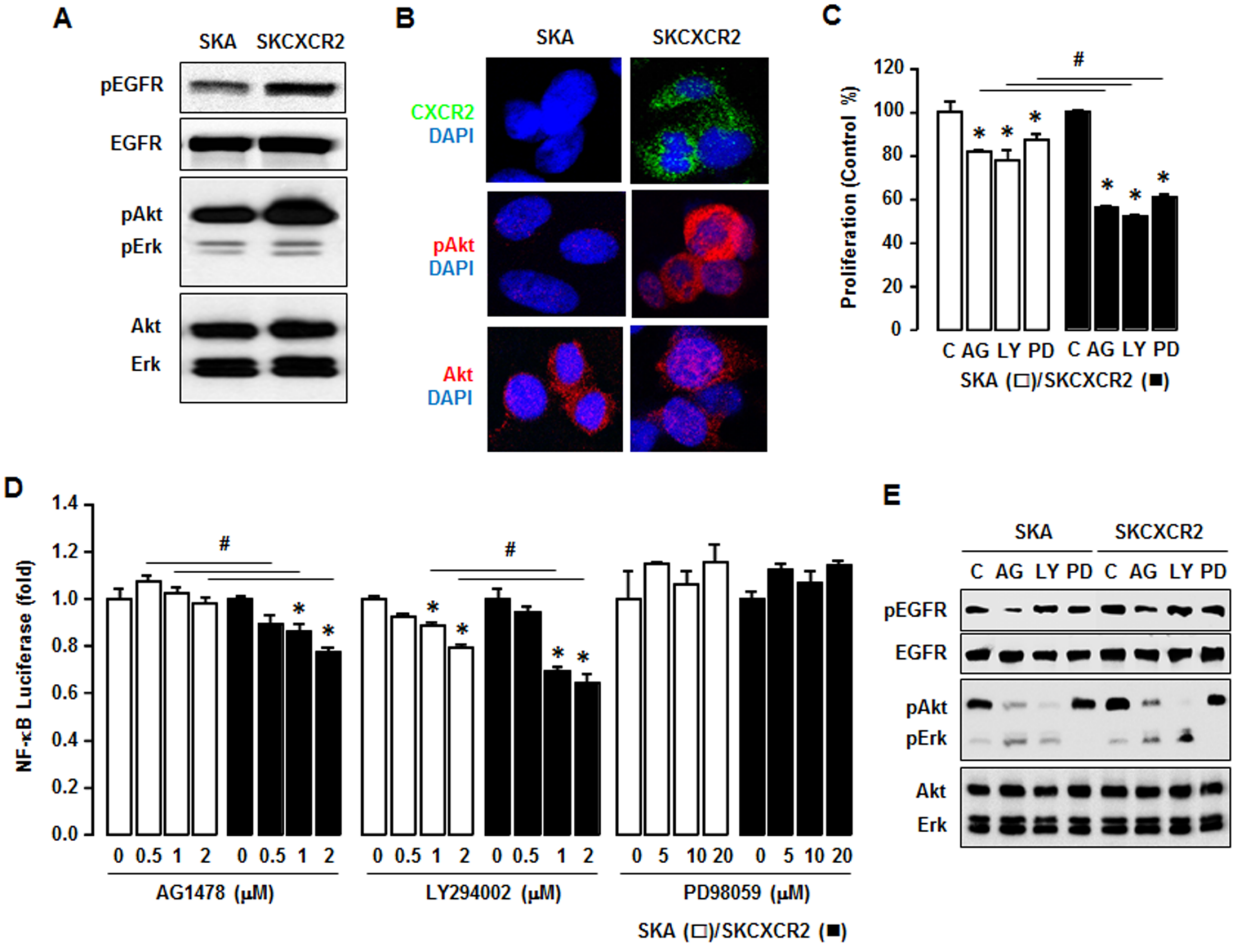
CXCR2 transactivates EGFR which contributes to NF-κB signaling via Akt activation. (A) Comparison of EGFR activation in SKA and SKCXCR2 cells. Whole cell lysates were prepared and Western blots carried out using antibodies specific to EGFR, Akt, Erk and the phosphorylated forms (pEGFR, pAkt and pErk). The non-phosphorylated forms were used as loading controls. (B) Representative immunofluorescent staining patterns indicating Akt activation and CXCR2 protein expression levels in SKA and SKCXCR2 cells. (C) Comparative effects of AG-1478, LY294002 and PD98059 on cell proliferation in SKA and SKCXCR2 cells. Cells were incubated with vehicle (Control), AG-1478 (AG, 2 µM), LY294002 (LY, 2 µM) or PD98059 (PD, 20 µM) for 48 h. The cell proliferation assay was performed using MTT and values were normalized to untreated controls. * and # (p≤0.05) when compared to Controls (C) and SKA cells, respectively, by Student’s *t*-test. (D) Dose-dependent effects of EGFR downstream inhibitors on NF-κB luciferase activities in SKA and SKCXCR2 cells. After transfection with NF-κB luciferase vector overnight, cells were treated with AG-1478 (EGFR inhibitor, 0, 0.5, 1 and 2 µM), LY294002 (Akt inhibitor, 0, 0.5, 1 and 2 µM) or PD98059 (Erk inhibitor, 0, 5, 10 and 20 µM) for 4 h. * and # (p≤0.05) when compared to Controls (0 h) and SKA cells, respectively, by Student’s *t*-test. All experiments were performed at least in triplicate and data are shown as mean ± S.E. (E) Confirmation of specific inhibitors on EGFR, Akt and Erk activation in SKA and SKCXCR2 cells. Cells were treated with AG-1478 (2 µM), LY294002 (2 µM) and PD98059 (20 µM) for 4 h. Whole cell lysates were prepared and a western blot was carried out using antibodies specific to EGFR, Akt, Erk and their phosphorylated forms (pEGFR, pAkt and pErk). Non-phosphorylated forms were used as loading controls.

Since it was shown that CXCL1 can induce proliferation in epithelial ovarian cancer cells by transactivation of EGFR [27], we compared EGFR transactivation in SKA and SKCXCR2 cells. SKCXCR2 cells had a greater level of phosphorylated EGFR, resulting to higher Akt activation, but there was little effect on pErk levels (Figure 4A). Confocal imaging revealed that SKCXCR2 cells had more phosphorylated Akt as compared to SKA cells (Figure 4B). Since Akt and Erk pathways relate to cell survival and proliferation, we checked the comparative effects of PI3K/Akt or Erk inhibitors on cell proliferation in SKA and SKCXCR2 cells. Although AG-1478, LY294002 and PD98059 attenuated cell proliferation in both SKA and SKCXCR2 cells, their effects on proliferation were far greater in SKCXCR2 cells (Figure 4C). We then determined if EGFR downstream inhibitors affected NF-κB promoter activity in SKA and SKCXCR2 cells. AG-1478, a specific EGFR kinase inhibitor, had no significant effect on NF-κB promoter activity in SKA cells, but it attenuated the activity in CXCR2 positive cells in a dose-dependent manner (Figure 4D). Although LY294002, a highly selective PI3K inhibitor that blocks Akt activation, attenuated NF-κB promoter activity in both cell types in a dose-dependent manner, it had a greater inhibitory effect on this activity in SKCXCR2 cells. Interestingly, PD98059, a specific Erk inhibitor, had no significant effect on NF-κB promoter activity in either cell type (Figure 4D). We confirmed the effects of specific inhibitors on EGFR, Akt and Erk activation (Figure 4E).

### CXCL1 Enhances NF-κB Activation in CXCR2 Expressing Cells Which Increases CXCL1 Promoter Activity via an NF-κB Site

**Figure 5:**
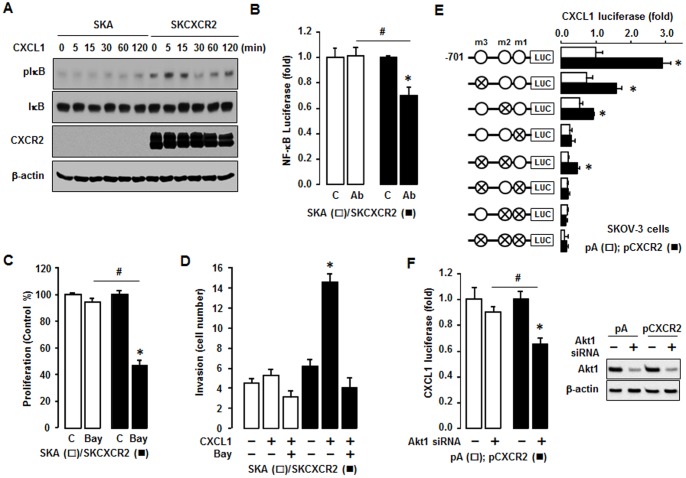
CXCL1 enhances NF-κB activation and CXCR2 increases CXCL1 promoter activity via NF-κB site. (A) Comparative effects of CXCL1 on NF-κB activation in SKA and SKCXCR2 cells. Cells were treated with CXCL1 (100 ng/ml) and results examined in a time-dependent manner. Whole cell lysates were prepared and Western blots carried out using antibodies specific to IκB, phosphorylated IκB (pIκB) and CXCR2. β-actin was used as a loading control. (B) Effect of CXCL1/2/3 antibody on NF-κB luciferase activities in SKA and SKCXCR2 cells. After transfection using the NF-κB luciferase vector overnight, cells were incubated with normal IgG (C) and the CXCL1/2/3 antibody (Ab, 1∶100 dilution)) for 4 h. (C) Effect of Bay11-7082 on cell proliferation in SKA and SKCXCR2 cells. Cells in A-B were incubated with vehicle (C) or Bay11-7082 (Bay, 2 µM) for 48 h. The cell proliferation assay was performed using MTT and values were normalized to untreated controls. * and # in 5B and 5C (p ≤ 0.05) when compared to Control (C) and SKA cells, respectively, by the paired Student’s *t*-test. (D) Inhibitory effects of Bay11-7082 (2 µM) on CXCL1-induced cell invasion in SKA and SKCXCR2 cells. * (p≤0.05) when compared to Control (C) and SKA cells, respectively, by the paired Student’s *t*-test. (E) Effect of CXCR2 on luciferase activity of mouse CXCL1 promoter in parental SKOV-3 cells. Site-directed mutants were generated from the KC701LUC using primers with mutant κB sites (termed m1, m2 and m3 indicates mutation site): −615/−585 mutant κB site (KC701LUCm3), −102/−71 mutant κB site (KC701LUCm2) and −83/−52 mutant κB site (KC701LUCm1). Additional mutants of κB sites were generated as KC701LUCm3m2, KC701LUCm3m1, KC701LUCm2m1, and KC701LUCm3m2m1. After transfection with CXCL1 luciferase vector overnight, a luciferase assay was performed. Results were normalized to the activity of internal control (pRL-TK vector) and expressed as a fold increase compared to empty vector (pA) controls. * (p≤0.05) when compared to its own control by Student’s *t*-test. Cross circles indicate κB site mutants. pA  =  empty vector transfection; pCXCR2  =  CXCR2 vector transfection. (F) Effect of Akt1 siRNA on CXCL1 promoter activity in parental SKOV-3 cells with empty vector or CXCR2 transfection. After transfection with CXCL1 luciferase vector and siRNAs for control and Akt1 (final concentration: 10 nM) overnight, a luciferase assay was performed. Results were normalized to total protein concentrations and expressed as a fold increase compared to each control. All experiments were performed at least in triplicate and all data are shown as mean ± S.E. * and # (p ≤ 0.05) when compared to Control siRNA and pA transfected cells, respectively, by Student’s *t*-test. We confirmed knockdown of Akt1 (main Akt isoform in parental SKOV-3 cells) by using Akt1 siRNA. Whole cell lysates were prepared and a western blot was carried out using Akt1 specific antibody. β-actin was used as a loading control.

To clarify involvement of NF-κB signaling in the CXCL1-CXCR2 axis, we tested the comparative effects of added CXCL1 on NF-κB activation in SKA and SKCXCR2 cells. CXCL1 produced more phosphorylated IκB in SKCXCR2 cells compared to SKA cells (Figure 5A). In addition, a CXCL1/2/3 antibody had no effects on NF-κB promoter activity in SKA cells but significantly decreased this activity in SKCXCR2 cells (Figure 5B). Based on the involvement of NF-κB in the CXCL1-CXCR2 axis, we compared effects of Bay11-7082, a specific NF-κB inhibitor, on cell proliferation in SKA and SKCXCR2 cells. Bay11-7082 had no effect on cell proliferation in SKA cells but significantly decreased proliferation in SKCXCR2 cells (Figure 5C). The inhibition was greatest in SKCXCR2 cells, probably because of the higher activation of NF-κB in these cells (Figures 2A-C). Furthermore, we compared the effects of Bay11-7082 on a CXCL1-induced cell invasion. Although CXCL1 had a small effect on cell invasion in SKA cells, the differences were not significant (Figure 5D). On the other hand, SKCXCR2 cells had at least a doubling of cell invasion numbers in response to CXCL1 compared to controls (Figure 5D). Bay11-7082 also blocked the CXCL1-induced cell invasion in SKCXCR2 cells (Figure 5D), indicating involvement of NF-κB signaling.

Next, we tested if transient transfection of CXCR2 into parental human SKOV-3 cells affected the activity of the CXCL1 promoter in an NF-κB-dependent manner. The CXCL1 promoter (KC701LUC) contains three NF-κB sites. Mutants of each κB site (termed m1, m2 and m3) were prepared as described previously [23]. The results show that the CXCL1 luciferase activities of KC701LUC and the κB site mutations m2 and m3 were increased in CXCR2 transfected cells compared to cells transfected with empty vector (pA) (Figure 5E). On the other hand, mutations of the proximal m1 κB site alone (and when in combination with the mutants KC701LUCm3, KC701LUCm2 and KC701LUCm3m2) were not responsive to enhanced CXCR2 expression (Figure 5E). These patterns were similar as described by interleukin-1 as the NF-κB activator in mouse granulaosa cells [23]. This finding indicates that the proximal κB site is essential for regulation of the CXCL1 promoter activity in response to CXCR2-mediated NF-κB and that the other two κB sites support induction of promoter activity. Furthermore, we tested involvement of Akt on CXCR2-mediated NF-κB signaling in CXCL1 promoter activity. We used a commercial siRNA of Akt1 (a main Akt isoform in SKOV-3 cells) to knockdown Akt1. Akt1 siRNA had no significant effect on CXCL1 promoter activity in CXCR2 negative cells, but it attenuated the activity in CXCR2 positive cells (Figure 5F).

### Comparison of CXCR2-driven Cancer Progression in OVA Versus OVCXCR2 Cells

**Figure 6:**
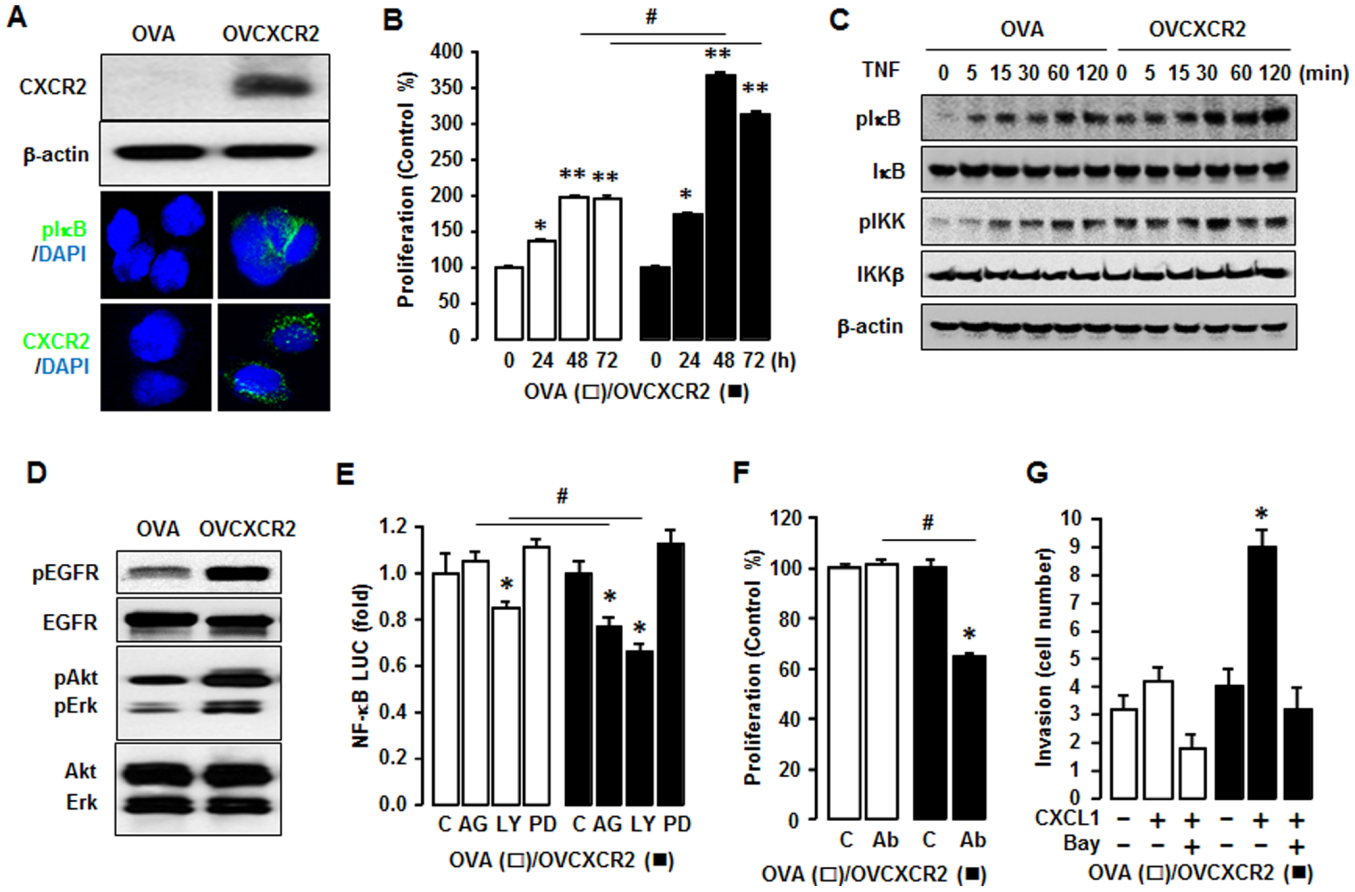
Confirmation of CXCR2-potentiated NF-κB signaling in OVCAR-3 cells. (A) CXCR2 protein expression in OVA versus OVCXCR2 cells. Western blot and immunofluorescent staining were carried out using antibodies specific to CXCR2 and β-actin as a loading control. (B) Comparison of growth rates in OVA and OVCXCR2 cells. Cells were incubated for 0, 24, 48 and 72 h and growth rates normalized to 0 h densities in each cell line. Experiments were performed in triplicate and all data are shown as mean ± S.E. * and ** (p≤0.05) in each group by ANOVA and Tukey’s pairwise comparisons. # (p≤0.05) between OVA and OVCXCR2 cells by Student’s *t*-test. (C) Effect of TNF (10 ng/ml) effects over time (0-120 min) on NF-κB activation in OVA and OVCXCR2 cells. Whole cell lysates were prepared and Western blots carried out using antibodies specific to IκB, IKK and their phosphorylated forms (pIκB and pIKK). β-actin was used as a loading control. (D) Comparison of EGFR activation in OVA and OVCXCR2 cells. Whole cell lysates were prepared and Western blots carried out using antibodies specific to EGFR, Akt, Erk and the phosphorylated forms (pEGFR, pAkt and pErk). The non-phosphorylated forms were used as loading controls. (E) Effects of EGFR downstream inhibitors on NF-κB luciferase activity in OVA and OVCXCR2 cells. After transfection with NF-κB luciferase vector overnight, cells were treated with vehicle (C), AG-1478 (AG, 2 µM), LY294002 (LY, 2 µM) or PD98059 (PD, 20 µM) for 4 h. (F) Effect of CXCL1/2/3 pan specific antibody for neutralization on cell proliferation in OVA and OVCXCR2 cells. Cells were incubated with normal IgG (C) and antibody (1∶100 dilution) for 48 h. The cell proliferation assay was performed using MTT and values were normalized to untreated controls. (G) Inhibitory effects of Bay11-7082 (2 µM) on CXCL1-induced cell invasion in OVA and OVCXCR2 cells. All experiments were performed at least in triplicate and data are shown as mean ± S.E. * and # (p≤0.05) as calculated by Student’s *t*-test.

To exclude an SKOV-3 cell-type specific response, we confirmed our data by generating another CXCR2 positive (OVCXCR2) versus a negative (OVA) cell line using parental OVCAR-3 ovarian cancer cells which either entirely lack or have trace amounts of CXCR2 [6]. The OVCAR-3 cell line was purchased from the American Type Culture Collection (Manassas, VA). OVCXCR2 cells expressed CXCR2 protein and immunofluorescent staining revealed that like SKCXCR2 cells (Figures 1A and 1B), OVCXCR2 cells had more phosphorylated IκB (Figure 6A) than OVA cells. The growth rates in OVCXCR2 vs. OVA cells (Figure 6B) were similar to those observed in SKCXCR2 vs. SKA cells (Figure 1C). OVCXCR2 cells also had more phosphorylated IκB in response to TNF (Figure 6C) as observed in SKCXCR2 cells (Figure 2C). OVCXCR2 cells had a greater level of phosphorylated EGFR, resulting to higher Akt and Erk activations (Figure 6D). AG-1478 and LY294002 reduced more NF-κB luciferase activity in OVCXCR2 cells whereas PD98059 had no effect (Figure 6E) as demonstrated in SKCXCR2 cells (Figure 4D). Also, a CXCL1/2/3 antibody blocked cell proliferation in OVCXCR2 cells as compared to OVA cells (Figure 6F). Bay11-7082 blocked the CXCL1-induced cell invasion in OVCXCR2 cells (Figure 6G) as observed in SKCXCR2 cells (Figure 5D). Because SKCXCR2 cells increased CXCR2 ligands such as CXCL1 and CXCL2 (Figure 2D), we confirmed that OVCXCR2 also increased CXCR2 ligands such as CXCL1-3 and 6 when compared to OVA cells (Figure S1). Additionally OVCXCR2 increased CCL2, probably resulting from a CXCR2-mediated NF-κB activation on κB sites in the CCL2 promoter [5]. Also CXCL1 activated more IκB in OVCXCR2 cells compared to OVA cells (Figure S2) as observed in SKCXCR2 cells (Figure 5A).

### CXCR2 shRNA Inhibits CXCR2-driven Cancer Progression in SKCXCR2 Cells

**Figure 7:**
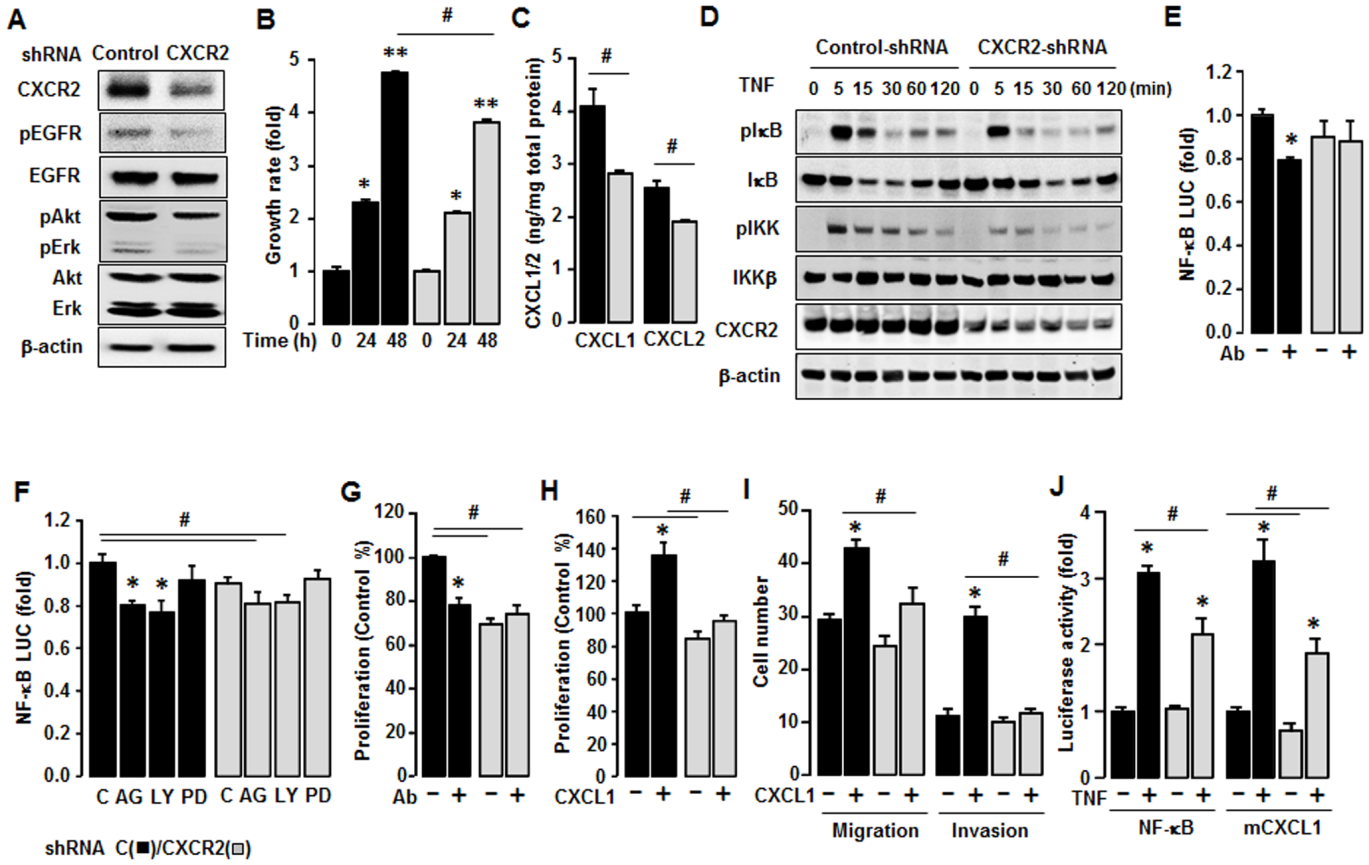
Inhibitory effect of CXCR2 shRNA on CXCR2-deriven cancer progression in SKCXCR2 cells. (A) Knockdown of CXCR2 protein expression and comparison of EGFR activation after transfection of Control and CXCR2 shRNA in SKCXCR2 cells. After transfection with shRNAs for control and CXCR2 for 72 h, a Western blot was carried out using antibodies specific to CXCR2, EGFR, Akt, Erk and the phosphorylated forms (pEGFR, pAkt and pErk). The non-phosphorylated forms and β-actin were used as loading controls. (B) Comparison of growth rates in Control (black bars) and CXCR2 shRNA (gray bars) transfected SKCXCR2 cells. Cells were incubated for 0, 24 and 48 h and growth rates normalized to 0 h densities in each cell line. Experiments were performed in triplicate and all data are shown as mean ± S.E. * and ** (p≤0.05) in each group by ANOVA and Tukey’s pairwise comparisons. # (p≤0.05) between Control and CXCR2 shRNA transfected cells by Student’s *t*-test. (C) Cellular CXCL1 and CXCL2 concentrations in Control and CXCR2 shRNA transfected cells. Whole cell lysates were prepared, an ELISA carried out using antibodies specific to CXCL1 and CXCL2 and values normalized to total protein. (D) Effect of TNF (10 ng/ml) over time (0–120 min) on NF-κB activation in Control and CXCR2 shRNA transfected cells. Whole cell lysates were prepared and Western blots carried out using antibodies specific to IκB, IKK and their phosphorylated forms (pIκB and pIKK). β-actin was used as a loading control. (E) Effect of CXCL1/2/3 antibody on NF-κB luciferase activities in Control and CXCR2 shRNA transfected cells. After transfection of shRNA for 48 h followed by transfection of NF-κB luciferase vector overnight, cells were incubated with normal IgG and the CXCL1/2/3 antibody (Ab, 1∶100 dilution) for 4 h. (F) Effects of EGRF downstream inhibitors on NF-κB luciferase activity in Control and CXCR2 shRNA transfected cells. After transfection of shRNA for 48 h followed by transfection of NF-κB luciferase vector overnight, cells were treated with vehicle (C), AG-1478 (AG, 2 µM), LY294002 (LY, 2 µM) or PD98059 (PD, 20 µM) for 4 h. (G) Effect of CXCL1/2/3 antibody on cell proliferation in Control and CXCR2 shRNA transfected cells. Cells were incubated with normal IgG and CXCL1/2/3 antibody (Ab, 1∶100 dilution) for 48 h. The cell proliferation assay was performed using MTT and values were normalized to untreated controls. (H) Effect of CXCL1 on cell proliferation in Control and CXCR2 shRNA transfected cells for 48 h incubation. (I) Comparison of CXCL1-induced cell migration and invasion in Control and CXCR2 shRNA transfected cells. (J) Effect of TNF on NF-κB and mCXCL1 promoter luciferase activities in Control and CXCR2 shRNA transfected cells. After transfection of shRNA for 48 h followed by transfection of NF-κB or CXCL1 promoter luciferase vector overnight, cells were treated with TNF (10 ng/ml) for 4 h. All experiments were performed at least in triplicate and data are shown as mean ± S.E. * and # (p≤0.05) as calculated by Student’s *t*-test.

To solidify the impact of NF-κB signaling on CXCR2-driven cancer progression, we employed CXCR2 shRNA to knockdown CXCR2 protein expression in SKCXCR2 cells and confirmed an inhibitory effect of CXCR2 shRNA. CXCR2 shRNA transfected cells knockdowned CXCR2 protein, resulting to reduced EGFR, Akt and Erk activation (Figure 7A) as compared to Control shRNA. Growth rates in CXCR2 shRNA transfected cells were similar for the first 24 h of culture but by 48 h were reduced, as compared to Control shRNA cells (Figure 7B). Also CXCR2 shRNA transfected cells produced less CXCL1 and CXCL2 proteins than Control shRNA cells (Figures 7C). In response to TNF, CXCR2 shRNA transfected cells had lesser phosphorylated IKK and IκB as compared to Control shRNA cells (Figure 7D). In addition, a CXCL1/2/3 antibody had no effects on NF-κB promoter activity in CXCR2 shRNA transfected cells but significantly decreased this activity in Control shRNA cells (Figure 7E). AG-1478 and LY294002 reduced NF-κB luciferase activity at a lesser degree in CXCR2 shRNA transfected cells as compared to Control shRNA cells and PD98059 had no effect in both cells (Figure 7F). Also, a CXCL1/2/3 antibody had no significant effects on cell proliferation in CXCR2 shRNA transfected cells as compared to Control shRNA cells (Figure 7G). Addition of CXCL1 had less effects on cell proliferation in CXCR2 shRNA transfected cells (Figure 7H). CXCR2 shRNA transfected cells had a reduced CXCL1-induced cell migration and invasion as compared to Control shRNA cells (Figure 7I). CXCR2 shRNA transfected cells also had a reduced TNF-induced NF-κB luciferase activity and had decreased basal and TNF-induced levels of CXCL1 promoter activity (Figure 7J).

## Discussion

A primary finding of this study is that CXCR2-driven cancer progression involves upregulation of its own ligands such as CXCL1 and CXCL2 by potentiating NF-κB activation via EGFR-transactivated Akt signaling followed by accelerated ovarian cancer cell proliferation, migration and invasion. TNF appears to have different proliferative characteristics, depending on the cells involved. As an example, although TNF inhibited proliferation in ovarian UT-OC-2 carcinoma cells [28], it had a proliferative effect in ovarian MDAH 2774 cancer cells [29]. Ovarian UT-OC-2 carcinoma cells had no NF-κB activation in respond to TNF whereas ovarian MDAH 2774 cancer cells induced TNF-activated NF-κB [28]–[29], indicating a critical role of NF-κB signaling on cell proliferation. Interestingly, saxatilin, a snake venom shown to inhibit TNF-induced proliferation in ovarian cancer cells, was found to block the TNF effect by suppressing CXCL8 expression [29]. This fact supports the concept that chemokines are involved in TNF-induced cell proliferation. Also, our previous studies have reported that TNF primarily induces CXCR2 ligands (CXCL1-3 and CXCL8) in ovarian cancer cells [5]–[6]. In the current study, TNF increased cell proliferation to a greater extent in CXCR2 positive compared to CXCR2 negative cells (Figure 1D). In spite of a significant difference in the G0-G1 phase in CXCR2 positive and negative cells (Figure 1E), TNF had no significant effects on cell cycle related genes except for a >2-fold increase of GADD45α in CXCR2 positive cells (Table S1). Interestingly, GADD45α has been found to have dual functions as both a promoter in Myc-driven breast cancer and a suppressor in Ras-driven breast cancer [30]. Although protecting melanoma cells from ultraviolet B-induced apoptosis [31] and cell death in neuron cells [32], GADD45α functions as a mediator of retinoid-induced apoptosis in ovarian carcinoma cells [24]. Disruption of GADD45α both promotes cell migration and invasion in endothelial cells [25] and mouse embryonic fibroblasts [26]. Because GADD45 protein is well known as a stress sensor [33], TNF-induced GADD45α is likely to be the cellular response to a TNF stress reaction instead of serving as a promoter of cell proliferation in CXCR2 positive cells.

Because NF-κB is the primary signaling pathway for TNF functions, we examined NF-κB signaling in CXCR2 negative and positive cells. CXCR2 positive cells exhibited higher levels of NF-κB activation in both the basal and TNF-induced states (Figures 2A, 2C and 6C). Knockdown of CXCR2 in CXCR2 positive cells decreased TNF-induced NF-κB activation (Figure 7D and 7J). Several prior reports have indirectly suggested the presence of a positive relationship between NF-κB signaling and the CXCR2 axis. For instance, the CXCR2 antagonist, SCH-527123, was able to decrease phosphorylation of NF-κB in colorectal cancer cells [34] while in ovarian cancer cells, CXCR2 stimulated angiogenesis by a process thought to also involve NF-κB [9]. In addition, neutrophils from severely NF-κB deficient mice [c-Rel(−/−)NF-κB1(−/−)RelA(+/−)] express higher levels of CXCR2 [35], which may serve as a compensatory mechanism for the NF-κB deficiency.

Because CXCR2 ligands such as CXCL1-3, and 5–8 [22] contain κB sites in their promoters [5]–[6], it is possible that CXCR2-mediated NF-κB activation is able to modulate the chemokine network, which in turn, alters cellular functional events. Among the CXCR2 ligands in this study, SKCXCR2 cells highly induced CXCL1 and CXCL2 compared to levels seen in SKA cells (Figures 2D, 3A and 3B). Knockdown of CXCR2 in SKCXCR2 cells decreased CXCL1 and CXCL2 production (Figure 7C). In case of OVCXCR2 cells, CXCL1-3 and 6 were induced as a CXCR2 ligands (Figure S1). Although CXCL1 has been reported to suppress malignancy by limiting prostate tumor metastasis and reinforcing growth arrest [36], many reports indicate that CXCL1 promotes cancer progression. For instance, CXCL1 depletion reduced the migration and invasion of gastric cancer cells [15] and an anti-CXCL1 antibody inhibited growth of human pancreatic cancer cells [37]. CXCL1/2 reportedly mediates breast cancer metastasis [38] and esophageal cancer cell proliferation [39]. CXCL1 also induced proliferation in epithelial ovarian cancer cells [27]. In addition to cancer cells, CXCL1 caused endothelial cell proliferation, tube formation, and migration [40]. Consistent with these reports, CXCR2 positive cells in this study led to a greater increase in proliferation, migration, invasion and colony formation (Figures 1C, 3F-3H, and 6B). Knockdown of CXCR2 in SKCXCR2 cells decreased CXCL1-induced proliferation, migration and invasion (Figure 7H and 7I). Furthermore, a CXCL1/2/3 antibody or an NF-κB inhibitor (Bay11-7082) was more inhibitory with regard to cell proliferation in CXCR2 positive cells (Figures 3D, 5C and 6F).

In these studies, we found that CXCR2 positive cells transactivated more EGFR followed by increased Akt activation than negative cells (Figures 4A, 4B and 6D). Thus knockdown of CXCR2 in SKCXCR2 cells decreased EGFR-activated signaling (Figure 7A). AG-1478, a specific EGFR inhibitor, and LY294002, an Akt blocker via PI3K inhibition, attenuated NF-κB promoter activity in CXCR2 positive cells whereas PD98059, a specific Erk inhibitor, had no effect (Figure 4D and 6E). On the other hand, knockdown of CXCR2 in SKCXCR2 cells attenuated effects of AG-1478 and LY294002 on NF-κB promoter activity (Figure 7F). Erk activation was relatively low when compared to Akt activation (Figure 4A, 4E and 6D), probably resulting to slight effects of PD98059 in this model system. Interestingly PD98059 had increased trend on NF-κB promoter activity (Figure 4D and 6E). These findings indicate that CXCR2-mediated EGFR transactivation contributes to NF-κB potentiation through Akt activation rather than Erk activation. Prior studies showed that blockade of Akt2 decreased IKKα phosphorylation, NF-κB nuclear translocation and cell migration in prostate cancer cells [41]. This fact supports in part the involvement of Akt in CXCR2-mediated NF-κB signaling as described by our results. Furthermore, because an NF-κB inhibitor attenuated CXCL1-induced cell invasion in CXCR2 positive cells (Figure 5D and 6G), our results suggest that the CXCL1-CXCR2 axis may accelerate cancer progression by potentiating NF-κB signaling.

In addition to NF-κB, CXCR2-mediated signaling could involve Akt and/or Erk activation. Prior studies indicate that the CXCR2 antagonist, SCH-527123, decreased Erk and Akt activation in colorectal cancer cells [34] and that CXCR2 knockdown reduced Erk activation in ovarian cancer cells [9]. Inhibition of Erk also blocked CXCL8-induced cell proliferation in non-small cell lung cancer cells [42]. Other investigators reported that a CXCL1-induced cell proliferation in ovarian cancer cells was related to transactivation of EGFR [24]. CXCL1 also enhanced potassium currents via activation of NF-κB in sensory neurons [43]. In our previous report, the mouse CXCL1 promoter (containing three κB sites) had a proximal κB site that served as a critical regulatory element and two other κB sites that served as supportive elements in mouse granulosa cells [23]. We demonstrated here that CXCR2 transiently-transfected human (SKOV-3) ovarian cancer cells similarly increased CXCL1 promoter activities via a critical proximal κB site (Figure 5E). Also knockdown of CXCR2 in SKCXCR2 cells reduced both basal and TNF-induced levels of CXCL1 promoter activity (Figure 7J). This fact further emphasizes the potentiation of NF-κB signaling in the CXCL1-CXCR2 axis. In addition, attenuation of Akt1 siRNA on CXCR2-induced CXCL1 promoter activity (Figure 5F) indicates the involvement of Akt activation.

In one exceptional report, CXCL1 overexpression acted as a suppressor of malignancy by limiting the escape of prostate tumor cells from the primary tumor and reinforcing growth arrest [36]. However, in many cases CXCL1 appears to act as autocrine or paracrine growth factor. Thus CXCL1 has been associated with tumor size, tumor stage, invasion, metastasis and survival in colorectal cancer patients [44]. Blocking CXCL1 signaling improved chemotherapy efficacy by diminishing metastasis in breast cancer [38]. In addition, an inverse association between CXCL1 and recurrence-free survival was observed in colorectal cancer patients [21]. Therefore, based on these functional roles of CXCL1 and CXCR2-mediated signaling in cancer progression, NF-κB potentiation is likely to play a central role in the CXCL1-CXCR2 axis.

**Figure 8:**
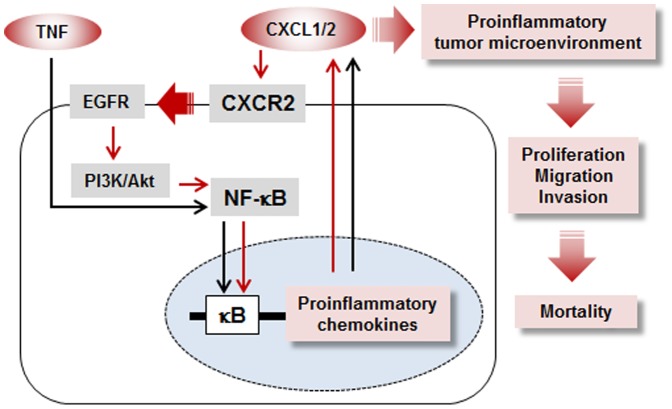
Proposed mechanisms by which CXCR2-driven cancer progression involves proinflammatory chemokines CXCL1/2 in CXCR2 positive ovarian cancer cells. TNF, a proinflammatory cytokine abundantly expressed in ovarian cancer, induces NF-κB activation followed by secretion of proinflammatory chemokines such as CXCL1-3 and CXCL8, all of which contain κB sites in their promoters (black line). In particular, these CXCR2 ligands (CXCL1-3 and CXCL8) respond to CXCR2 positive cancer cells (but not CXCR2 negative cells), thereby potentiating NF-κB activation via EGFR-transactivated Akt signaling. The enhanced NF-κB activity induces further CXCL1/2 secretion (red lines) reinforcing the proinflammatory tumor microenvironment to promote ovarian cancer progression (such as peritoneal tumor dissemination and massive ascites) followed by higher mortality rates. Therefore, inflammatory conditions can further intensify NF-κB activation in CXCR2 expressing ovarian cancer cells leading to acceleration of cancer progression.

In summary, CXCR2 expressing ovarian cancer cells potentiated NF-κB activation via EGFR-transactivated Akt signaling to induce CXCL1/2 secretion as an autocrine or paracrine growth factor. This in turn enhanced ovarian cancer progression including cell proliferation, migration and invasion events by increasing the proinflammatory tumor microenvironment (Figure 8). Activation of the CXCL1/2-CXCR2 axis could therefore augment the clinical features of ovarian cancer such as peritoneal tumor dissemination and ascites leading to higher mortality rates (Figure 8). Therefore inhibition of the CXCL1/2-CXCR2 axis may be an effective preventive or therapeutic action against CXCR2-driven ovarian cancer progression.

## Supporting Information

Figure S1 **Chemokine profile comparisons in OVCXCR2 relative to OVA cells.** After isolating total RNA, a human chemokine PCR array was performed. The dotted line indicates a 2-fold increase; those with a >2-fold increase and average cycle threshold <30 are recognized as induced chemokines (*). In this case, significant increases (OVCXCR2 versus OVA) were seen in CCL2, and CXCL 1-3 and 6.
(TIF)

Figure S2 **Comparative effects of CXCL1 on NF-κB activation in OVA and OVCXCR2 cells.** Cells were treated with CXCL1 (100 ng/ml) and results examined in a time-dependent manner. Whole cell lysates were prepared and Western blots carried out using antibodies specific to IκB, phosphorylated IκB (pIκB) and CXCR2. As a loading control, β-actin was used.
(TIF)

Table S1 **Comparative effects of TNF on cell-cycle related genes between SKA and SKCXCR2 cells as determined by PCR array.**
(DOCX)

## References

[1] Chobanian N, Dietrich CS (2008) Ovarian cancer. Surg Clin North Am 88: 285–299. 18381114 10.1016/j.suc.2007.12.002

[2] Dobrzycka B, Terlikowski SJ, Kowalczuk O, Kinalski M (2009) Circulating levels of TNF-α and its soluble receptors in the plasma of patients with epithelial ovarian cancer. Eur Cytokine Netw 20: 131–134. 19825522 10.1684/ecn.2009.0161

[3] Macciò A, Madeddu C (2012) Inflammation and ovarian cancer. Cytokine 58: 133–147. 22349527 10.1016/j.cyto.2012.01.015

[4] Szlosarek PW, Grimshaw MJ, Kulbe H, Wilson JL, Wilbanks GD, et al (2006) Expression and regulation of tumor necrosis factor alpha in normal and malignant ovarian epithelium. Mol Cancer Ther 5: 382–390. 16505113 10.1158/1535-7163.MCT-05-0303

[5] Son DS, Parl AK, Rice VM, Khabele D (2007) Keratinocyte chemoattractant (KC)/human growth-regulated oncogene (GRO) chemokines and pro-inflammatory chemokine networks in mouse and human ovarian epithelial cancer cells. Cancer Biol Ther 6: 1302–1312. 17712227 10.4161/cbt.6.8.4506

[6] Son DS, Kabir SM, Dong YL, Lee E, Adunyah SE (2012) Inhibitory effect of tumor suppressor p53 on proinflammatory chemokine expression in ovarian cancer cells by reducing proteasomal degradation of IκB. PLoS One 7: e51116. 23300534 10.1371/journal.pone.0051116 PMC3534106

[7] Balkwill FR (2012) The chemokine system and cancer. J Pathol 226: 148–157. 21989643 10.1002/path.3029

[8] Singh R, Lillard JW Jr, Singh S (2011) Chemokines: key players in cancer progression and metastasis. Front Biosci 3: 1569–1582. 10.2741/246 PMC3760587 21622291

[9] Yang G, Rosen DG, Liu G, Yang F, Guo X, et al (2010) CXCR2 promotes ovarian cancer growth through dysregulated cell cycle, diminished apoptosis, and enhanced angiogenesis. Clin Cancer Res 16: 3875–3886. 20505188 10.1158/1078-0432.CCR-10-0483 PMC2930833

[10] Saintigny P, Massarelli E, Lin S, Ahn YH, Chen Y, et al (2013) CXCR2 expression in tumor cells is a poor prognostic factor and promotes invasion and metastasis in lung adenocarcinoma. Cancer Res 73: 571–582. 23204236 10.1158/0008-5472.CAN-12-0263 PMC3548940

[11] Han L, Jiang B, Wu H, Wang X, Tang X, et al (2012) High expression of CXCR2 is associated with tumorigenesis, progression, and prognosis of laryngeal squamous cell carcinoma. Med Oncol 29: 2466–2472. 22274915 10.1007/s12032-011-0152-1

[12] Ewington L, Taylor A, Sriraksa R, Horimoto Y, Lam EW, et al (2012) The expression of interleukin-8 and interleukin-8 receptors in endometrial carcinoma. Cytokine 59: 417–422. 22626766 10.1016/j.cyto.2012.04.036

[13] Bondurant KL, Lundgreen A, Herrick JS, Kadlubar S, Wolff RK, et al (2013) Interleukin genes and associations with colon and rectal cancer risk and overall survival. Int J Cancer 132: 905–915. 22674296 10.1002/ijc.27660 PMC3470814

[14] Liu Z, Yang L, Xu J, Zhang X, Wang B (2011) Enhanced expression and clinical significance of chemokine receptor CXCR2 in hepatocellular carcinoma. J Surg Res 166: 241–246. 20018298 10.1016/j.jss.2009.07.014

[15] Cheng WL, Wang CS, Huang YH, Tsai MM, Liang Y, et al (2011) Lin KH. Overexpression of CXCL1 and its receptor CXCR2 promote tumor invasion in gastric cancer. Ann Oncol 22: 2267–2276. 21343381 10.1093/annonc/mdq739

[16] Shen H, Schuster R, Lu B, Waltz SE, Lentsch AB (2006) Critical and opposing roles of the chemokine receptors CXCR2 and CXCR3 in prostate tumor growth. Prostate 66: 1721–1728. 16941672 10.1002/pros.20476

[17] Keane MP, Belperio JA, Xue YY, Burdick MD, Strieter RM (2004) Depletion of CXCR2 inhibits tumor growth and angiogenesis in a murine model of lung cancer. J Immunol 172: 2853–2860. 14978086 10.4049/jimmunol.172.5.2853

[18] Mestas J, Burdick MD, Reckamp K, Pantuck A, Figlin RA, et al (2005) The role of CXCR2/CXCR2 ligand biological axis in renal cell carcinoma. J Immunol 175: 5351–5357. 16210641 10.4049/jimmunol.175.8.5351

[19] Jamieson T, Clarke M, Steele CW, Samuel MS, Neumann J, et al (2012) Inhibition of CXCR2 profoundly suppresses inflammation-driven and spontaneous tumorigenesis. J Clin Invest 122: 3127–3144. 22922255 10.1172/JCI61067 PMC3428079

[20] Lee YS, Choi I, Ning Y, Kim NY, Khatchadourian V, et al (2012) Interleukin-8 and its receptor CXCR2 in the tumour microenvironment promote colon cancer growth, progression and metastasis. Br J Cancer 106: 1833–1841. 22617157 10.1038/bjc.2012.177 PMC3364111

[21] Oladipo O, Conlon S, O'Grady A, Purcell C, Wilson C, et al (2011) The expression and prognostic impact of CXC-chemokines in stage II and III colorectal cancer epithelial and stromal tissue. Br J Cancer 104: 480–487. 21285972 10.1038/sj.bjc.6606055 PMC3049559

[22] Olson TS, Ley K (2002) Chemokines and chemokine receptors in leukocyte trafficking. Am J Physiol Regul Integr Comp Physiol 283: R7–R28. 12069927 10.1152/ajpregu.00738.2001

[23] Son DS, Roby KF (2006) Interleukin-1α-induced chemokines in mouse granulosa cells: impact on keratinocyte chemoattractant chemokine, a CXC subfamily. Mol Endocrinol 20: 2999–3013. 16825293 10.1210/me.2006-0001

[24] Jiang T, Soprano DR, Soprano KJ (2007) GADD45A is a mediator of CD437 induced apoptosis in ovarian carcinoma cells. J Cell Physiol 212: 771–779. 17474084 10.1002/jcp.21073

[25] Yang F, Zhang W, Li D, Zhan Q (2013) Gadd45α suppresses tumor angiogenesis via inhibition of the mTOR/STAT3 pathway. J Biol Chem 288: 6552–6560. 23329839 10.1074/jbc.M112.418335 PMC3585088

[26] Shan Z, Li G, Zhan Q, Li D (2012) Gadd45a inhibits cell migration and invasion by altering the global RNA expression. Cancer Biol Ther 13: 1112–1122. 22825327 10.4161/cbt.21186 PMC3461817

[27] Bolitho C, Hahn MA, Baxter RC, Marsh DJ (2010) The chemokine CXCL1 induces proliferation in epithelial ovarian cancer cells by transactivation of the epidermal growth factor receptor. Endocr Relat Cancer 17: 929–940. 20702723 10.1677/ERC-10-0107

[28] Seppänen M, Lin L, Saarinen R, Punnonen R, Vihko KK (2008) Regulation of ovarian UT-OC-2 carcinoma cells by cytokines: effects on cell proliferation, activation of transcription factors and apoptosis. Acta Obstet Gynecol Scand 87: 902–909. 18720042 10.1080/00016340802283921

[29] Kim DS, Jang YJ, Jeon OH, Kim DS (2007) Saxatilin inhibits TNF-α-induced proliferation by suppressing AP-1-dependent IL-8 expression in the ovarian cancer cell line MDAH 2774. Mol Immunol 44: 1409–1416. 16806476 10.1016/j.molimm.2006.05.001

[30] Tront JS, Huang Y, Fornace AJ Jr, Hoffman B, Liebermann DA (2010) Gadd45α functions as a promoter or suppressor of breast cancer dependent on the oncogenic stress. Cancer Res 70: 9671–9681. 21098706 10.1158/0008-5472.CAN-10-2177 PMC3199142

[31] Fayolle C, Pourchet J, Caron de Fromentel C, Puisieux A, Doré JF, et al (2008) Gadd45a activation protects melanoma cells from ultraviolet B-induced apoptosis. J Invest Dermatol 128: 196–202. 17703175 10.1038/sj.jid.5700963

[32] Lin CR, Yang CH, Huang CE, Wu CH, Chen YS, et al (2011) GADD45A protects against cell death in dorsal root ganglion neurons following peripheral nerve injury. J Neurosci Res 89: 689–699. 21337369 10.1002/jnr.22589

[33] Liebermann DA, Tront JS, Sha X, Mukherjee K, Mohamed-Hadley A, et al (2011) Gadd45 stress sensors in malignancy and leukemia. Crit Rev Oncog 16: 129–140. 22150313 10.1615/critrevoncog.v16.i1-2.120 PMC3268054

[34] Ning Y, Labonte MJ, Zhang W, Bohanes PO, Gerger A, et al (2012) The CXCR2 antagonist, SCH-527123, shows antitumor activity and sensitizes cells to oxaliplatin in preclinical colon cancer models. Mol Cancer Ther 11: 1353–1364. 22391039 10.1158/1535-7163.MCT-11-0915

[35] von Vietinghoff S, Asagiri M, Azar D, Hoffmann A, Ley K (2010) Defective regulation of CXCR2 facilitates neutrophil release from bone marrow causing spontaneous inflammation in severely NF-κB-deficient mice. J Immunol 185: 670–678. 20519647 10.4049/jimmunol.1000339 PMC2891140

[36] Benelli R, Stigliani S, Minghelli S, Carlone S, Ferrari N (2013) Impact of CXCL1 overexpression on growth and invasion of prostate cancer cell. Prostate 73: 941–951. 23334998 10.1002/pros.22640

[37] Takamori H, Oades ZG, Hoch OC, Burger M, Schraufstatter IU (2000) Autocrine growth effect of IL-8 and GROalpha on a human pancreatic cancer cell line, Capan-1. Pancreas 21: 52–56. 10881932 10.1097/00006676-200007000-00051

[38] Acharyya S, Oskarsson T, Vanharanta S, Malladi S, Kim J, et al (2012) A CXCL1 paracrine network links cancer chemoresistance and metastasis. Cell 150: 165–178. 22770218 10.1016/j.cell.2012.04.042 PMC3528019

[39] Wang B, Hendricks DT, Wamunyokoli F, Parker MI (2006) A growth-related oncogene/CXC chemokine receptor 2 autocrine loop contributes to cellular proliferation in esophageal cancer. Cancer Res 66: 3071–3077. 16540656 10.1158/0008-5472.CAN-05-2871

[40] Agarwal A, Tressel SL, Kaimal R, Balla M, Lam FH, et al (2010) Identification of a metalloprotease-chemokine signaling system in the ovarian cancer microenvironment: implications for antiangiogenic therapy. Cancer Res 70: 5880–5890. 20570895 10.1158/0008-5472.CAN-09-4341 PMC2917243

[41] Kuo PL, Shen KH, Hung SH, Hsu YL (2012) CXCL1/GROα increases cell migration and invasion of prostate cancer by decreasing fibulin-1 expression through NF-κB/HDAC1 epigenetic regulation. Carcinogenesis 33: 2477–2487. 23027620 10.1093/carcin/bgs299

[42] Luppi F, Longo AM, de Boer WI, Rabe KF, Hiemstra PS (2007) Interleukin-8 stimulates cell proliferation in non-small cell lung cancer through epidermal growth factor receptor transactivation. Lung Cancer 56: 25–33. 17175059 10.1016/j.lungcan.2006.11.014

[43] Yang RH, Strong JA, Zhang JM (2009) NF-κB mediated enhancement of potassium currents by the chemokine CXCL1/growth related oncogene in small diameter rat sensory neurons. Mol Pain 5: 26. 19476648 10.1186/1744-8069-5-26 PMC2698898

[44] Ogata H, Sekikawa A, Yamagishi H, Ichikawa K, Tomita S, et al (2010) GROα promotes invasion of colorectal cancer cells. Oncol Rep 24: 1479–1486. 21042742

