# Supplementary figures and images for: CXCR2-Driven Ovarian Cancer Progression Involves Upregulation of Proinflammatory Chemokines by Potentiating NF-κB Activation via EGFR-Transactivated Akt Signaling
Source: PLoS One. 2013 Dec 20;8(12):e83789. doi: 10.1371/journal.pone.0083789 (PMC3869803; doi:10.1371/journal.pone.0083789)

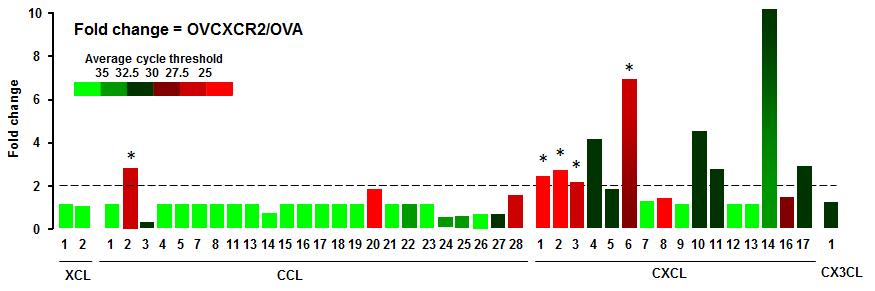

Supplement: Figure S1 — Chemokine profile comparisons in OVCXCR2 relative to OVA cells. After isolating total RNA, a human chemokine PCR array was performed. The dotted line indicates a 2-fold increase; those with a >2-fold increase and average cycle threshold <30 are recognized as induced chemokines (*). In this case, significant increases (OVCXCR2 versus OVA) were seen in CCL2, and CXCL 1-3 and 6. (TIF) [file pone.0083789.s001.tif]

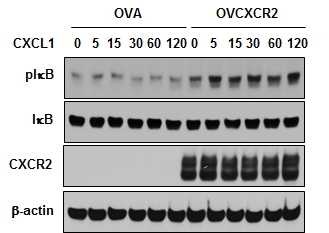

Supplement: Figure S2 — Comparative effects of CXCL1 on NF-κB activation in OVA and OVCXCR2 cells. Cells were treated with CXCL1 (100 ng/ml) and results examined in a time-dependent manner. Whole cell lysates were prepared and Western blots carried out using antibodies specific to IκB, phosphorylated IκB (pIκB) and CXCR2. As a loading control, β-actin was used. (TIF) [file pone.0083789.s002.tif]
