# Supplementary material for: CXCR2-Driven Ovarian Cancer Progression Involves Upregulation of Proinflammatory Chemokines by Potentiating NF-κB Activation via EGFR-Transactivated Akt Signaling
Source: PLoS One. 2013 Dec 20;8(12):e83789. doi: 10.1371/journal.pone.0083789 (PMC3869803; doi:10.1371/journal.pone.0083789)
Supplement: Table S1 — Comparative effects of TNF on cell-cycle related genes between SKA and SKCXCR2 cells as determined by PCR array. (DOCX) [file pone.0083789.s003.docx]

**Table S1. Comparative effects of TNF on cell-cycle related genes between SKA and SKCXCR2 cells as determined by PCR array**

| **Symbol** | **SKA-TNF** | **CXCR2C** | **CXCR2TNF** | **Level** | **Access No.** | **Description** |
| --- | --- | --- | --- | --- | --- | --- |
| ABL1 | 0.85 | 0.58 | 0.71 | H | NM_005157 | C-abl oncogene 1, non-receptor tyrosine kinase |
| ANAPC2 | 0.98 | 0.64 | 0.67 | H | NM_013366 | Anaphase promoting complex subunit 2 |
| ANAPC4 | 1.19 | 0.80 | 0.64 | H | NM_013367 | Anaphase promoting complex subunit 4 |
| DIRAS3 | 1.11 | 0.67 | 0.53 | L | NM_004675 | DIRAS family, GTP-binding RAS-like 3 |
| ATM | 0.91 | 1.18 | 1.21 | H | NM_000051 | Ataxia telangiectasia mutated |
| ATR | 1.12 | 0.94 | 0.62 | H | NM_001184 | Ataxia telangiectasia and Rad3 related |
| BAX | 0.88 | 0.67 | 0.92 | H | NM_004324 | BCL2-associated X protein |
| BCCIP | 0.98 | 1.15 | 1.49 | H | NM_016567 | BRCA2 and CDKN1A interacting protein |
| BCL2 | 1.14 | 0.84 | 1.18 | M | NM_000633 | B-cell CLL/lymphoma 2 |
| BIRC5 | 1.15 | 0.64 | 0.79 | H | NM_001168 | Baculoviral IAP repeat containing 5 |
| BRCA1 | 0.87 | 0.74 | 0.92 | H | NM_007294 | Breast cancer 1, early onset |
| BRCA2 | 0.82 | 1.46 | 1.70 | H | NM_000059 | Breast cancer 2, early onset |
| CCNB1 | 0.91 | **0.49** | **0.49** | H | NM_031966 | Cyclin B1 |
| CCNB2 | 1.03 | 0.51 | 0.56 | H | NM_004701 | Cyclin B2 |
| CCNC | 1.18 | 1.32 | 1.41 | H | NM_005190 | Cyclin C |
| CCND1 | 0.93 | 0.53 | 1.06 | H | NM_053056 | Cyclin D1 |
| CCND2 | 1.07 | 1.08 | 0.87 | N/D | NM_001759 | Cyclin D2 |
| CCNE1 | 0.83 | 0.76 | 0.94 | H | NM_001238 | Cyclin E1 |
| CCNF | 0.84 | **0.38** | 0.54 | H | NM_001761 | Cyclin F |
| CCNG1 | 0.90 | 1.33 | 1.17 | H | NM_004060 | Cyclin G1 |
| CCNG2 | 0.80 | **0.47** | **0.28** | H | NM_004354 | Cyclin G2 |
| CCNH | 1.07 | 1.02 | 1.27 | H | NM_001239 | Cyclin H |
| CCNT1 | 0.78 | 0.79 | 0.96 | H | NM_001240 | Cyclin T1 |
| CCNT2 | 0.76 | 0.57 | 0.62 | H | NM_001241 | Cyclin T2 |
| CDC16 | 0.96 | 1.18 | 1.10 | H | NM_003903 | Cell division cycle 16 homolog (S. cerevisiae) |
| CDK1 | 0.99 | 0.54 | 0.64 | H | NM_001786 | Cyclin-dependent kinase 1 |
| CDC20 | 0.82 | 0.60 | 0.76 | H | NM_001255 | Cell division cycle 20 homolog (S. cerevisiae) |
| CDC34 | 0.91 | 0.76 | 0.88 | H | NM_004359 | Cell division cycle 34 homolog (S. cerevisiae) |
| CDK2 | 1.05 | 0.79 | 0.85 | H | NM_001798 | Cyclin-dependent kinase 2 |
| CDK4 | 1.15 | 0.89 | 0.95 | H | NM_000075 | Cyclin-dependent kinase 4 |
| CDK5R1 | 0.82 | 0.50 | 0.72 | H | NM_003885 | Cyclin-dependent kinase 5, regulatory subunit 1 (p35) |
| CDK5RAP1 | 1.16 | 1.13 | 1.10 | H | NM_016408 | CDK5 regulatory subunit associated protein 1 |
| CDK6 | 1.19 | 1.09 | 1.87 | H | NM_001259 | Cyclin-dependent kinase 6 |
| CDK7 | 1.00 | 1.15 | 1.19 | H | NM_001799 | Cyclin-dependent kinase 7 |
| CDK8 | 1.03 | 0.64 | 0.99 | H | NM_001260 | Cyclin-dependent kinase 8 |
| CDKN1A | 1.23 | **0.25** | **0.29** | H | NM_000389 | Cyclin-dependent kinase inhibitor 1A (p21, Cip1) |
| CDKN1B | 0.81 | 0.62 | 0.56 | H | NM_004064 | Cyclin-dependent kinase inhibitor 1B (p27, Kip1) |
| CDKN2A | 1.09 | 1.33 | 1.25 | L | NM_000077 | Cyclin-dependent kinase inhibitor 2A (melanoma, p16, inhibits CDK4) |
| CDKN2B | 0.94 | 0.74 | 1.14 | L | NM_004936 | Cyclin-dependent kinase inhibitor 2B (p15, inhibits CDK4) |
| CDKN3 | 0.89 | 0.92 | 0.87 | H | NM_005192 | Cyclin-dependent kinase inhibitor 3 |
| CHEK1 | 0.91 | 0.57 | 0.86 | H | NM_001274 | CHK1 checkpoint homolog (S. pombe) |
| CHEK2 | 0.77 | 0.72 | 0.64 | H | NM_007194 | CHK2 checkpoint homolog (S. pombe) |
| CKS1B | 1.03 | 1.08 | 1.52 | H | NM_001826 | CDC28 protein kinase regulatory subunit 1B |
| CKS2 | 1.01 | 0.78 | 1.00 | H | NM_001827 | CDC28 protein kinase regulatory subunit 2 |
| CUL1 | 1.14 | 0.70 | 0.69 | H | NM_003592 | Cullin 1 |
| CUL2 | 0.94 | 1.08 | 0.99 | H | NM_003591 | Cullin 2 |
| CUL3 | 0.75 | 0.91 | 0.59 | H | NM_003590 | Cullin 3 |
| DDX11 | 0.78 | 0.68 | 0.67 | H | NM_004399 | DEAD/H (Asp-Glu-Ala-Asp/His) box polypeptide 11 |
| DNM2 | 0.86 | 0.92 | 0.74 | H | NM_004945 | Dynamin 2 |
| E2F4 | 0.97 | 1.03 | 1.40 | H | NM_001950 | E2F transcription factor 4, p107/p130-binding |
| GADD45A | 1.27 | 0.98 | **2.52** | H | NM_001924 | Growth arrest and DNA-damage-inducible, alpha |
| GTF2H1 | 1.07 | 1.33 | 1.09 | H | NM_005316 | General transcription factor IIH, polypeptide 1, 62kDa |
| GTSE1 | 1.17 | 0.66 | 0.87 | H | NM_016426 | G-2 and S-phase expressed 1 |
| HERC5 | 1.05 | 0.76 | 0.77 | H | NM_016323 | Hect domain and RLD 5 |
| HUS1 | 0.71 | 0.50 | 0.80 | H | NM_004507 | HUS1 checkpoint homolog (S. pombe) |
| KNTC1 | 0.84 | 0.82 | 0.72 | H | NM_014708 | Kinetochore associated 1 |
| KPNA2 | 0.82 | 0.57 | 0.58 | H | NM_002266 | Karyopherin alpha 2 (RAG cohort 1, importin alpha 1) |
| MAD2L1 | 1.02 | 0.92 | 1.03 | H | NM_002358 | MAD2 mitotic arrest deficient-like 1 (yeast) |
| MAD2L2 | 0.90 | 0.75 | 0.93 | H | NM_006341 | MAD2 mitotic arrest deficient-like 2 (yeast) |
| MCM2 | 0.77 | 0.76 | 1.05 | H | NM_004526 | Minichromosome maintenance complex component 2 |
| MCM3 | 0.74 | 1.02 | 1.70 | H | NM_002388 | Minichromosome maintenance complex component 3 |
| MCM4 | 0.98 | 0.83 | 0.99 | H | NM_005914 | Minichromosome maintenance complex component 4 |
| MCM5 | 0.99 | 0.63 | 1.10 | H | NM_006739 | Minichromosome maintenance complex component 5 |
| MKI67 | 1.08 | 0.76 | 0.95 | H | NM_002417 | Antigen identified by monoclonal antibody Ki-67 |
| MNAT1 | 0.99 | 0.53 | **0.43** | H | NM_002431 | Menage a trois homolog 1, cyclin H assembly factor (Xenopus laevis) |
| MRE11A | 0.78 | 0.78 | 0.67 | H | NM_005590 | MRE11 meiotic recombination 11 homolog A (S. cerevisiae) |
| NBN | 0.94 | 0.93 | 0.69 | H | NM_002485 | Nibrin |
| PCNA | 0.94 | 0.80 | 0.92 | H | NM_182649 | Proliferating cell nuclear antigen |
| RAD1 | 1.17 | 0.91 | 0.68 | H | NM_002853 | RAD1 homolog (S. pombe) |
| RAD17 | 1.10 | 0.82 | 0.80 | H | NM_002873 | RAD17 homolog (S. pombe) |
| RAD51 | 1.25 | 1.16 | 1.88 | M | NM_002875 | RAD51 homolog (S. cerevisiae) |
| RAD9A | 0.79 | 0.80 | 0.76 | H | NM_004584 | RAD9 homolog A (S. pombe) |
| RB1 | 0.73 | 0.96 | 1.07 | H | NM_000321 | Retinoblastoma 1 |
| RBBP8 | 1.17 | 0.99 | 1.17 | H | NM_002894 | Retinoblastoma binding protein 8 |
| RBL1 | 0.66 | **0.32** | **0.27** | H | NM_002895 | Retinoblastoma-like 1 (p107) |
| RBL2 | 0.91 | 0.62 | **0.45** | H | NM_005611 | Retinoblastoma-like 2 (p130) |
| RPA3 | 1.10 | 1.22 | 0.82 | H | NM_002947 | Replication protein A3, 14kDa |
| SERTAD1 | 1.15 | 0.96 | 1.22 | H | NM_013376 | SERTA domain containing 1 |
| SKP2 | 0.75 | 0.77 | 0.90 | H | NM_005983 | S-phase kinase-associated protein 2 (p45) |
| SUMO1 | 0.91 | 0.76 | 0.71 | H | NM_003352 | SMT3 suppressor of mif two 3 homolog 1 (S. cerevisiae) |
| TFDP1 | 0.91 | 1.13 | 1.20 | H | NM_007111 | Transcription factor Dp-1 |
| TFDP2 | 0.84 | 0.86 | 1.04 | H | NM_006286 | Transcription factor Dp-2 (E2F dimerization partner 2) |
| TP53 | 0.86 | 0.62 | 0.69 | H | NM_000546 | Tumor protein p53 |
| UBA1 | 1.00 | 0.94 | 0.97 | H | NM_003334 | Ubiquitin-like modifier activating enzyme 1 |

Fold changes were compared with Controls in which all SKA cell values were set at 1.00, after normalization of housekeeping genes such as actin and glyceraldehyde-3-phosphate dehydrogenase. Thus the 3 columns of ratios [SKA-TNF=TNF added to SKA cells; CXCR2C = control SKCXCR2 cells; and CXCR2-TNF = TNF added to SKCXCR2 cells) are reported relative to SKA values. Bold letters indicate above or below two-fold difference. Under Level, H = a high level (<30 average threshold cycles); L = a low level (30-35 cycles); M = a fluctuating level between L and H; and N/D (>35 cycles) = not determined.
